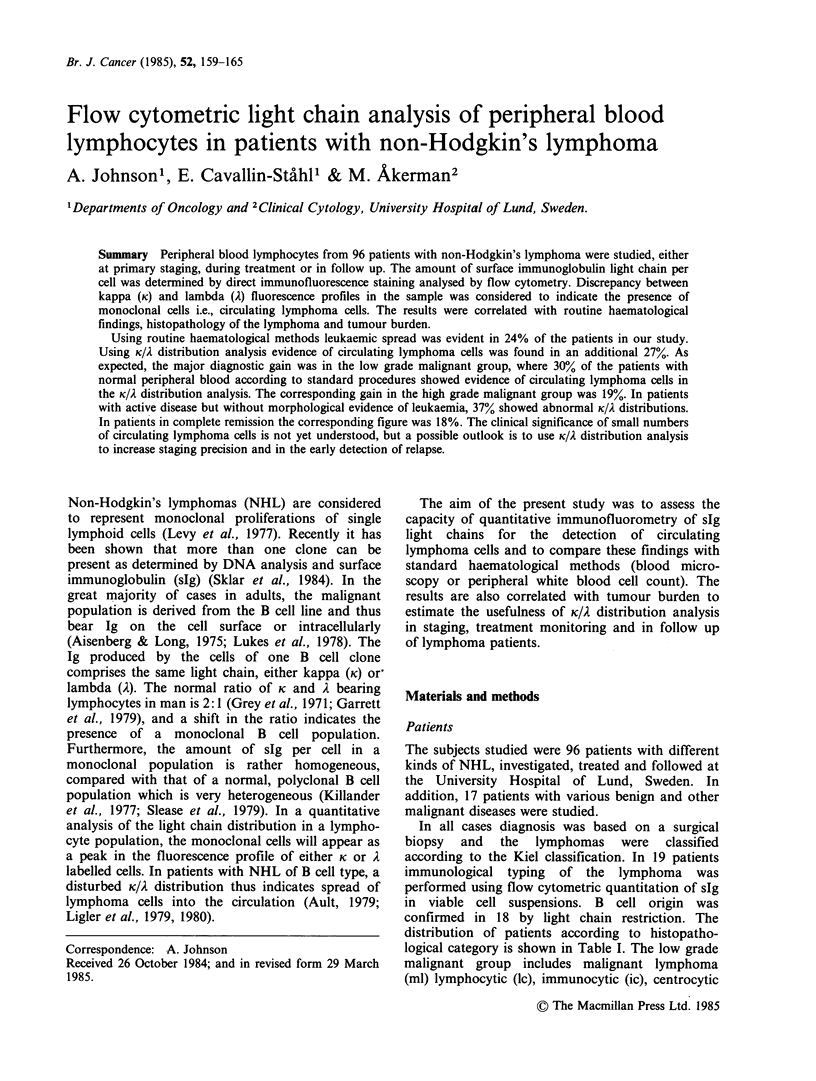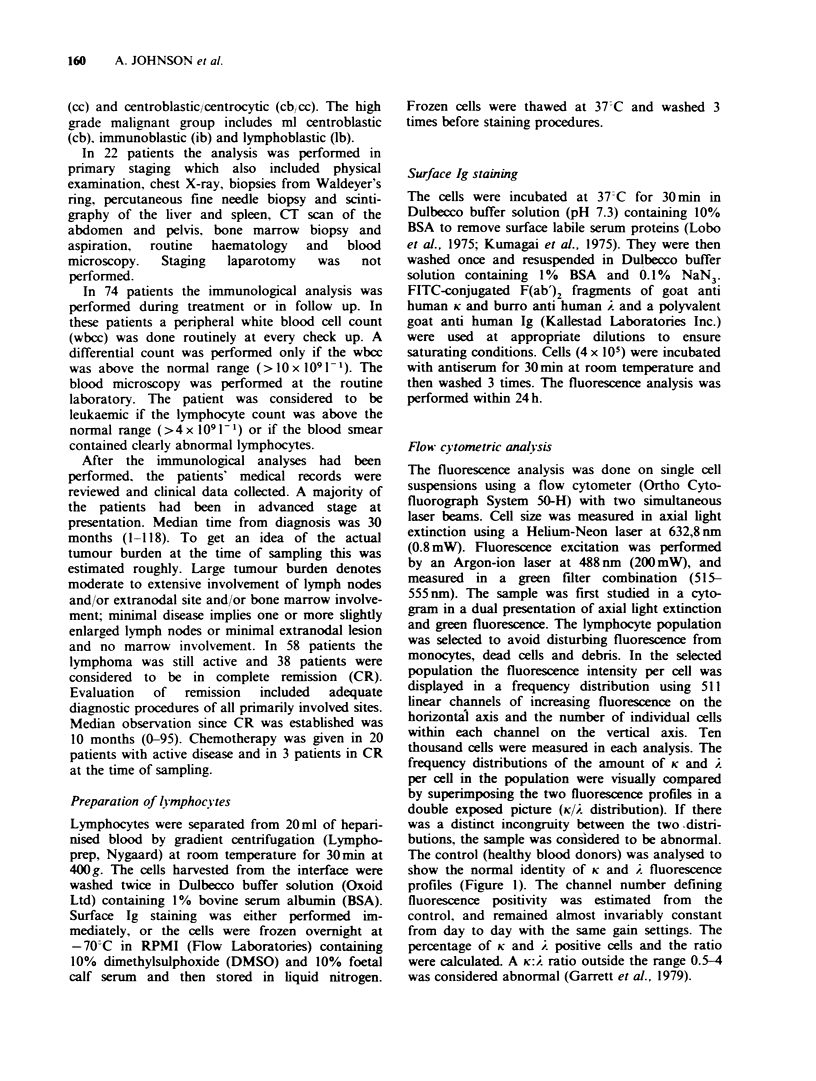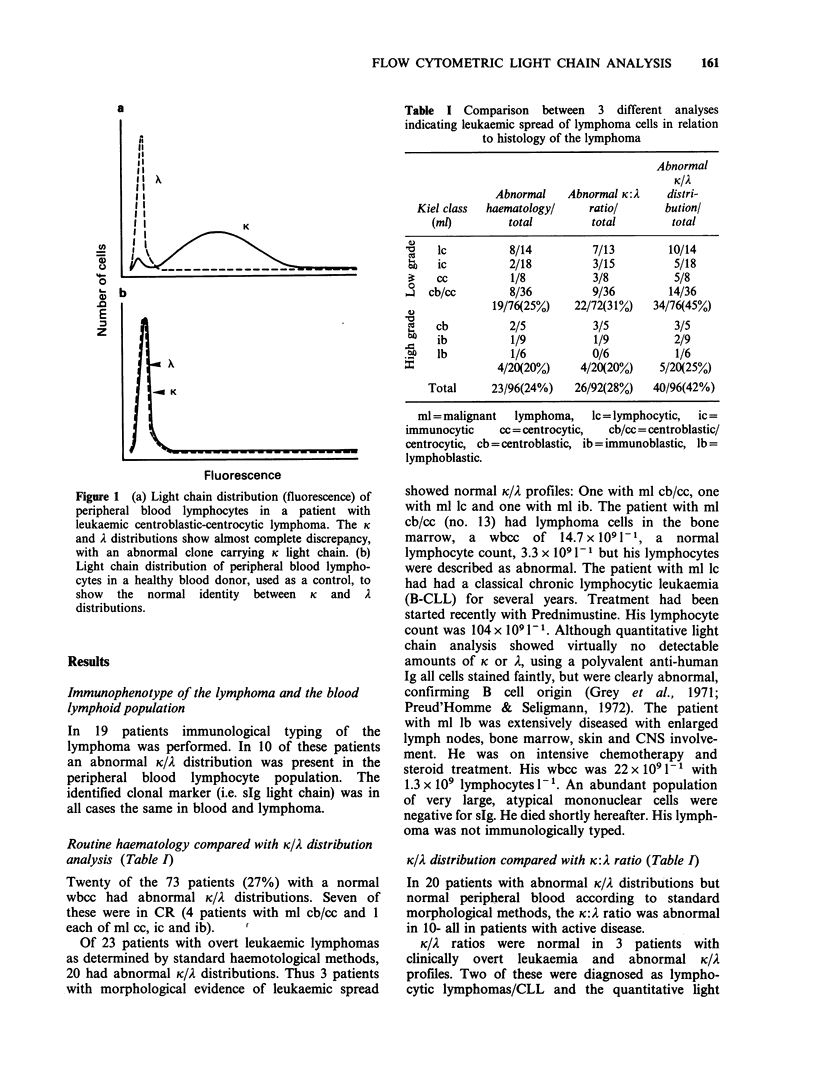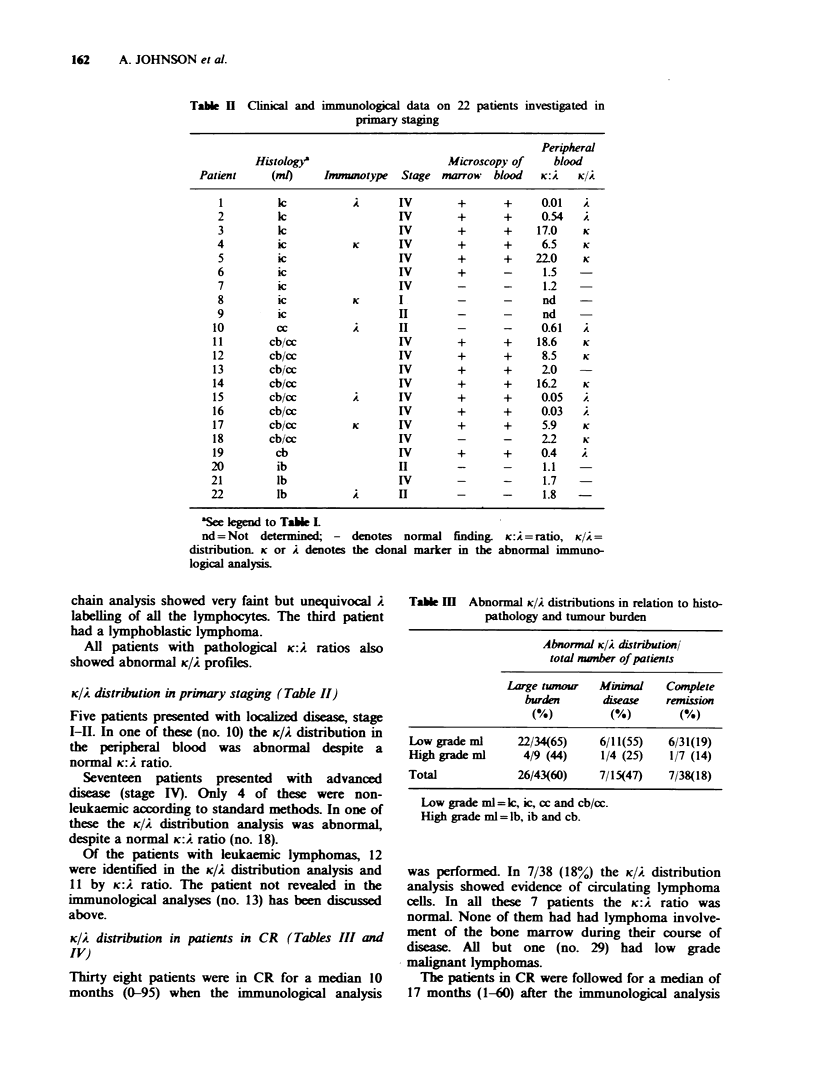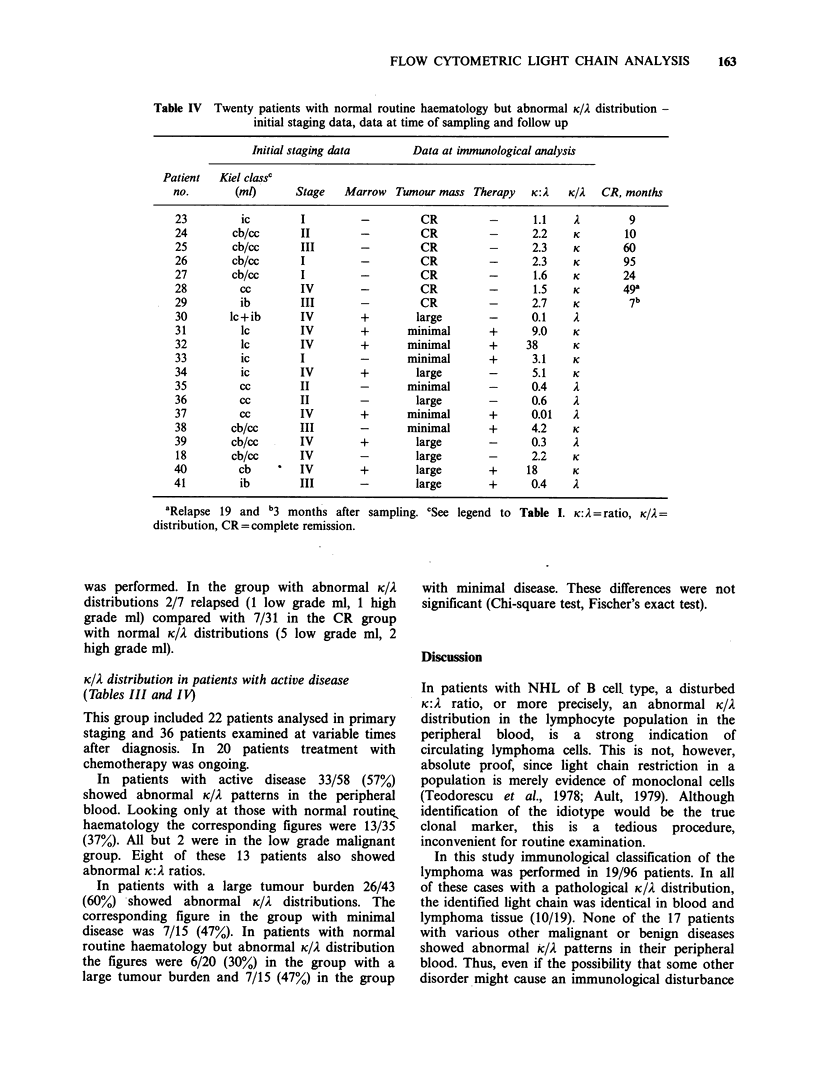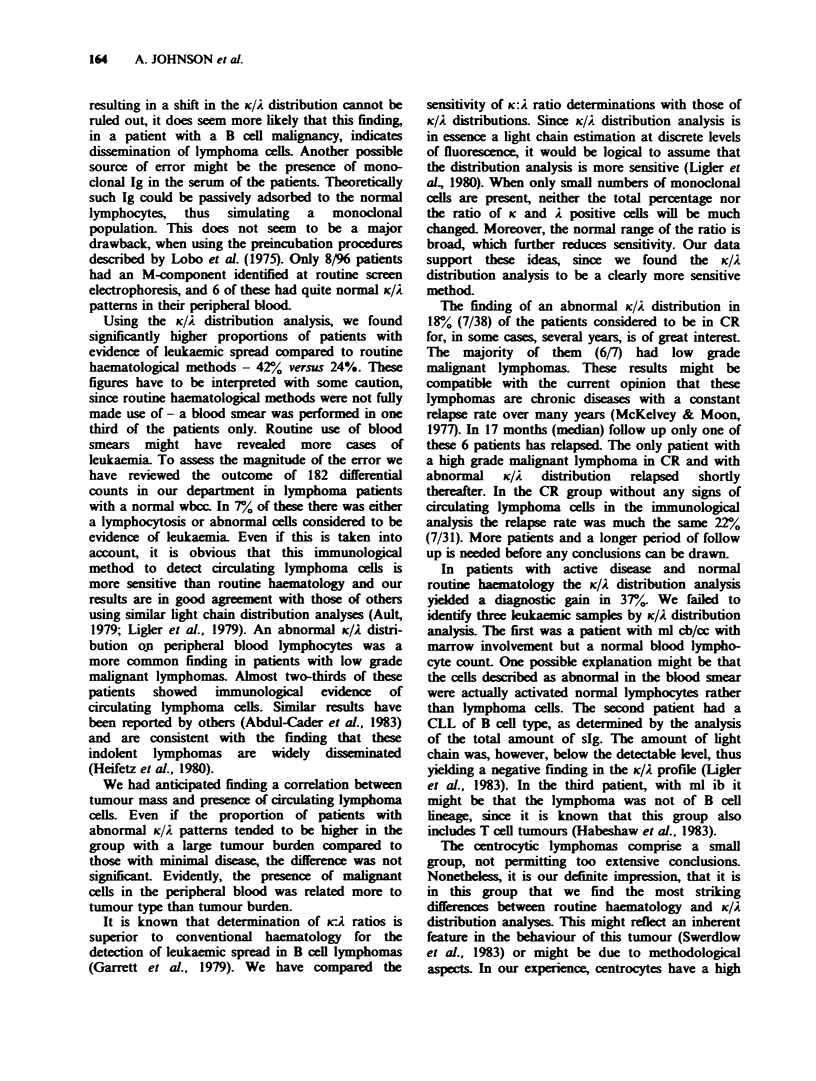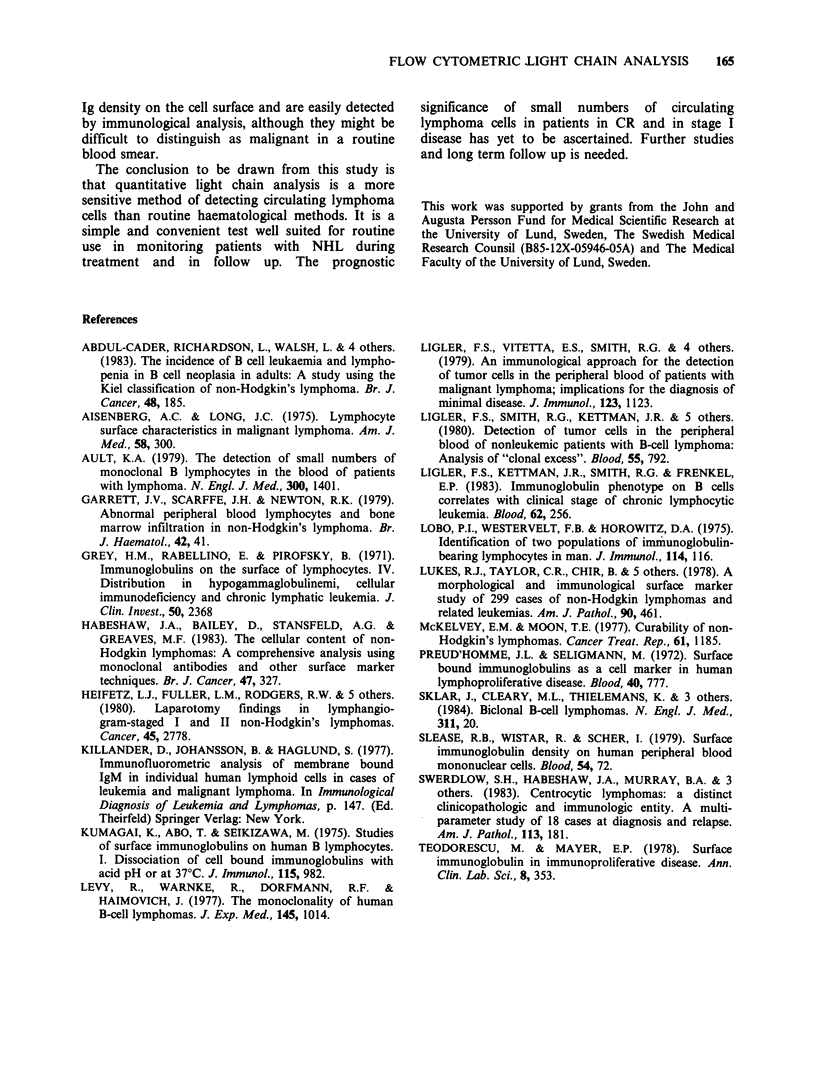# Flow cytometric light chain analysis of peripheral blood lymphocytes in patients with non-Hodgkin's lymphoma.

**DOI:** 10.1038/bjc.1985.172

**Published:** 1985-08

**Authors:** A. Johnson, E. Cavallin-Ståhl, M. Akerman

## Abstract

Peripheral blood lymphocytes from 96 patients with non-Hodgkin's lymphoma were studied, either at primary staging, during treatment or in follow up. The amount of surface immunoglobulin light chain per cell was determined by direct immunofluorescence staining analysed by flow cytometry. Discrepancy between kappa and lambda fluorescence profiles in the sample was considered to indicate the presence of monoclonal cells i.e., circulating lymphoma cells. The results were correlated with routine haematological findings, histopathology of the lymphoma and tumour burden. Using routine haematological methods leukaemic spread was evident in 24% of the patients in our study. Using kappa/lambda distribution analysis evidence of circulating lymphoma cells was found in an additional 27%. As expected, the major diagnostic gain was in the low grade malignant group, where 30% of the patients with normal peripheral blood according to standard procedures showed evidence of circulating lymphoma cells in the kappa/lambda distribution analysis. The corresponding gain in the high grade malignant group was 19%. In patients with active disease but without morphological evidence of leukaemia, 37% showed abnormal kappa/lambda distributions. In patients in complete remission the corresponding figure was 18%. The clinical significance of small numbers of circulating lymphoma cells is not yet understood, but a possible outlook is to use kappa/lambda distribution analysis to increase staging precision and in the early detection of relapse.


*Br. J. Cancer* (1985), **52**, 159–165

### Flow cytometric light chain analysis of peripheral blood lymphocytes in patients with non-Hodgkin's lymphoma

A. Johnson[1], E. Cavallin-Ståhl[1] & M. Åkerman[2]

[1]*Departments of Oncology and* [2]*Clinical Cytology, University Hospital of Lund, Sweden.*

**Summary** Peripheral blood lymphocytes from 96 patients with non-Hodgkin's lymphoma were studied, either at primary staging, during treatment or in follow up. The amount of surface immunoglobulin light chain per cell was determined by direct immunofluorescence staining analysed by flow cytometry. Discrepancy between kappa ($\kappa$) and lambda ($\lambda$) fluorescence profiles in the sample was considered to indicate the presence of monoclonal cells i.e., circulating lymphoma cells. The results were correlated with routine haematological findings, histopathology of the lymphoma and tumour burden.

Using routine haematological methods leukaemic spread was evident in 24% of the patients in our study. Using $\kappa/\lambda$ distribution analysis evidence of circulating lymphoma cells was found in an additional 27%. As expected, the major diagnostic gain was in the low grade malignant group, where 30% of the patients with normal peripheral blood according to standard procedures showed evidence of circulating lymphoma cells in the $\kappa/\lambda$ distribution analysis. The corresponding gain in the high grade malignant group was 19%. In patients with active disease but without morphological evidence of leukaemia, 37% showed abnormal $\kappa/\lambda$ distributions. In patients in complete remission the corresponding figure was 18%. The clinical significance of small numbers of circulating lymphoma cells is not yet understood, but a possible outlook is to use $\kappa/\lambda$ distribution analysis to increase staging precision and in the early detection of relapse.

Non-Hodgkin's lymphomas (NHL) are considered to represent monoclonal proliferations of single lymphoid cells (Levy *et al.*, 1977). Recently it has been shown that more than one clone can be present as determined by DNA analysis and surface immunoglobulin (sIg) (Sklar *et al.*, 1984). In the great majority of cases in adults, the malignant population is derived from the B cell line and thus bear Ig on the cell surface or intracellularly (Aisenberg & Long, 1975; Lukes *et al.*, 1978). The Ig produced by the cells of one B cell clone comprises the same light chain, either kappa ($\kappa$) or lambda ($\lambda$). The normal ratio of $\kappa$ and $\lambda$ bearing lymphocytes in man is 2:1 (Grey *et al.*, 1971; Garrett *et al.*, 1979), and a shift in the ratio indicates the presence of a monoclonal B cell population. Furthermore, the amount of sIg per cell in a monoclonal population is rather homogeneous, compared with that of a normal, polyclonal B cell population which is very heterogeneous (Killander *et al.*, 1977; Slease *et al.*, 1979). In a quantitative analysis of the light chain distribution in a lymphocyte population, the monoclonal cells will appear as a peak in the fluorescence profile of either $\kappa$ or $\lambda$ labelled cells. In patients with NHL of B cell type, a disturbed $\kappa/\lambda$ distribution thus indicates spread of lymphoma cells into the circulation (Ault, 1979; Ligler *et al.*, 1979, 1980).

Correspondence: A. Johnson
Received 26 October 1984; and in revised form 29 March 1985.

The aim of the present study was to assess the capacity of quantitative immunofluorometry of sIg light chains for the detection of circulating lymphoma cells and to compare these findings with standard haematological methods (blood microscopy or peripheral white blood cell count). The results are also correlated with tumour burden to estimate the usefulness of $\kappa/\lambda$ distribution analysis in staging, treatment monitoring and in follow up of lymphoma patients.

### Materials and methods

### *Patients*

The subjects studied were 96 patients with different kinds of NHL, investigated, treated and followed at the University Hospital of Lund, Sweden. In addition, 17 patients with various benign and other malignant diseases were studied.

In all cases diagnosis was based on a surgical biopsy and the lymphomas were classified according to the Kiel classification. In 19 patients immunological typing of the lymphoma was performed using flow cytometric quantitation of sIg in viable cell suspensions. B cell origin was confirmed in 18 by light chain restriction. The distribution of patients according to histopathological category is shown in Table I. The low grade malignant group includes malignant lymphoma (ml) lymphocytic (lc), immunocytic (ic), centrocytic

© The Macmillan Press Ltd. 1985

(cc) and centroblastic/centrocytic (cb/cc). The high grade malignant group includes ml centroblastic (cb), immunoblastic (ib) and lymphoblastic (lb).

In 22 patients the analysis was performed in primary staging which also included physical examination, chest X-ray, biopsies from Waldeyer's ring, percutaneous fine needle biopsy and scintigraphy of the liver and spleen, CT scan of the abdomen and pelvis, bone marrow biopsy and aspiration, routine haematology and blood microscopy. Staging laparotomy was not performed.

In 74 patients the immunological analysis was performed during treatment or in follow up. In these patients a peripheral white blood cell count (wbcc) was done routinely at every check up. A differential count was performed only if the wbcc was above the normal range ($>10\times10^9\,l^{-1}$). The blood microscopy was performed at the routine laboratory. The patient was considered to be leukaemic if the lymphocyte count was above the normal range ($>4\times10^9\,l^{-1}$) or if the blood smear contained clearly abnormal lymphocytes.

After the immunological analyses had been performed, the patients' medical records were reviewed and clinical data collected. A majority of the patients had been in advanced stage at presentation. Median time from diagnosis was 30 months (1–118). To get an idea of the actual tumour burden at the time of sampling this was estimated roughly. Large tumour burden denotes moderate to extensive involvement of lymph nodes and/or extranodal site and/or bone marrow involvement; minimal disease implies one or more slightly enlarged lymph nodes or minimal extranodal lesion and no marrow involvement. In 58 patients the lymphoma was still active and 38 patients were considered to be in complete remission (CR). Evaluation of remission included adequate diagnostic procedures of all primarily involved sites. Median observation since CR was established was 10 months (0–95). Chemotherapy was given in 20 patients with active disease and in 3 patients in CR at the time of sampling.

*Preparation of lymphocytes*

Lymphocytes were separated from 20 ml of heparinised blood by gradient centrifugation (Lymphoprep, Nygaard) at room temperature for 30 min at 400 *g*. The cells harvested from the interface were washed twice in Dulbecco buffer solution (Oxoid Ltd) containing 1% bovine serum albumin (BSA). Surface Ig staining was either performed immediately, or the cells were frozen overnight at −70°C in RPMI (Flow Laboratories) containing 10% dimethylsulphoxide (DMSO) and 10% foetal calf serum and then stored in liquid nitrogen. Frozen cells were thawed at 37°C and washed 3 times before staining procedures.

*Surface Ig staining*

The cells were incubated at 37°C for 30 min in Dulbecco buffer solution (pH 7.3) containing 10% BSA to remove surface labile serum proteins (Lobo *et al.*, 1975; Kumagai *et al.*, 1975). They were then washed once and resuspended in Dulbecco buffer solution containing 1% BSA and 0.1% $NaN_3$. FITC-conjugated $F(ab')_2$ fragments of goat anti human $\kappa$ and burro anti human $\lambda$ and a polyvalent goat anti human Ig (Kallestad Laboratories Inc.) were used at appropriate dilutions to ensure saturating conditions. Cells ($4\times10^5$) were incubated with antiserum for 30 min at room temperature and then washed 3 times. The fluorescence analysis was performed within 24 h.

*Flow cytometric analysis*

The fluorescence analysis was done on single cell suspensions using a flow cytometer (Ortho Cytofluorograph System 50-H) with two simultaneous laser beams. Cell size was measured in axial light extinction using a Helium-Neon laser at 632,8 nm (0.8 mW). Fluorescence excitation was performed by an Argon-ion laser at 488 nm (200 mW), and measured in a green filter combination (515–555 nm). The sample was first studied in a cytogram in a dual presentation of axial light extinction and green fluorescence. The lymphocyte population was selected to avoid disturbing fluorescence from monocytes, dead cells and debris. In the selected population the fluorescence intensity per cell was displayed in a frequency distribution using 511 linear channels of increasing fluorescence on the horizontal axis and the number of individual cells within each channel on the vertical axis. Ten thousand cells were measured in each analysis. The frequency distributions of the amount of $\kappa$ and $\lambda$ per cell in the population were visually compared by superimposing the two fluorescence profiles in a double exposed picture ($\kappa/\lambda$ distribution). If there was a distinct incongruity between the two distributions, the sample was considered to be abnormal. The control (healthy blood donors) was analysed to show the normal identity of $\kappa$ and $\lambda$ fluorescence profiles (Figure 1). The channel number defining fluorescence positivity was estimated from the control, and remained almost invariably constant from day to day with the same gain settings. The percentage of $\kappa$ and $\lambda$ positive cells and the ratio were calculated. A $\kappa$:$\lambda$ ratio outside the range 0.5–4 was considered abnormal (Garrett *et al.*, 1979).

**Figure 1** (a) Light chain distribution (fluorescence) of peripheral blood lymphocytes in a patient with leukaemic centroblastic-centrocytic lymphoma. The κ and λ distributions show almost complete discrepancy, with an abnormal clone carrying κ light chain. (b) Light chain distribution of peripheral blood lymphocytes in a healthy blood donor, used as a control, to show the normal identity between κ and λ distributions.

### Results

### *Immunophenotype of the lymphoma and the blood lymphoid population*

In 19 patients immunological typing of the lymphoma was performed. In 10 of these patients an abnormal κ/λ distribution was present in the peripheral blood lymphocyte population. The identified clonal marker (i.e. sIg light chain) was in all cases the same in blood and lymphoma.

### *Routine haematology compared with κ/λ distribution analysis (Table I)*

Twenty of the 73 patients (27%) with a normal wbcc had abnormal κ/λ distributions. Seven of these were in CR (4 patients with ml cb/cc and 1 each of ml cc, ic and ib).

Of 23 patients with overt leukaemic lymphomas as determined by standard haemotological methods, 20 had abnormal κ/λ distributions. Thus 3 patients with morphological evidence of leukaemic spread showed normal κ/λ profiles: One with ml cb/cc, one with ml lc and one with ml ib. The patient with ml cb/cc (no. 13) had lymphoma cells in the bone marrow, a wbcc of $14.7 \times 10^9 l^{-1}$, a normal lymphocyte count, $3.3 \times 10^9 l^{-1}$ but his lymphocytes were described as abnormal. The patient with ml lc had had a classical chronic lymphocytic leukaemia (B-CLL) for several years. Treatment had been started recently with Prednimustine. His lymphocyte count was $104 \times 10^9 l^{-1}$. Although quantitative light chain analysis showed virtually no detectable amounts of κ or λ, using a polyvalent anti-human Ig all cells stained faintly, but were clearly abnormal, confirming B cell origin (Grey *et al.*, 1971; Preud'Homme & Seligmann, 1972). The patient with ml lb was extensively diseased with enlarged lymph nodes, bone marrow, skin and CNS involvement. He was on intensive chemotherapy and steroid treatment. His wbcc was $22 \times 10^9 l^{-1}$ with $1.3 \times 10^9$ lymphocytes $l^{-1}$. An abundant population of very large, atypical mononuclear cells were negative for sIg. He died shortly hereafter. His lymphoma was not immunologically typed.

**Table I** Comparison between 3 different analyses indicating leukaemic spread of lymphoma cells in relation to histology of the lymphoma

| | *Kiel class (ml)* | *Abnormal haematology/ total* | *Abnormal κ:λ ratio/ total* | *Abnormal κ/λ distribution/ total* |
|---|---|---|---|---|
| Low grade | lc | 8/14 | 7/13 | 10/14 |
| | ic | 2/18 | 3/15 | 5/18 |
| | cc | 1/8 | 3/8 | 5/8 |
| | cb/cc | 8/36 | 9/36 | 14/36 |
| | | 19/76(25%) | 22/72(31%) | 34/76(45%) |
| High grade | cb | 2/5 | 3/5 | 3/5 |
| | ib | 1/9 | 1/9 | 2/9 |
| | lb | 1/6 | 0/6 | 1/6 |
| | | 4/20(20%) | 4/20(20%) | 5/20(25%) |
| | Total | 23/96(24%) | 26/92(28%) | 40/96(42%) |

ml = malignant lymphoma, lc = lymphocytic, ic = immunocytic cc = centrocytic, cb/cc = centroblastic/ centrocytic, cb = centroblastic, ib = immunoblastic, lb = lymphoblastic.

### *κ/λ distribution compared with κ:λ ratio (Table I)*

In 20 patients with abnormal κ/λ distributions but normal peripheral blood according to standard morphological methods, the κ:λ ratio was abnormal in 10- all in patients with active disease.

κ/λ ratios were normal in 3 patients with clinically overt leukaemia and abnormal κ/λ profiles. Two of these were diagnosed as lymphocytic lymphomas/CLL and the quantitative light

**Table II** Clinical and immunological data on 22 patients investigated in primary staging

| Patient | Histology[a] (ml) | Immunotype | Stage | Microscopy of marrow | Microscopy of blood | Peripheral blood κ:λ | Peripheral blood κ/λ |
|---|---|---|---|---|---|---|---|
| 1 | lc | λ | IV | + | + | 0.01 | λ |
| 2 | lc | | IV | + | + | 0.54 | λ |
| 3 | lc | | IV | + | + | 17.0 | κ |
| 4 | ic | κ | IV | + | + | 6.5 | κ |
| 5 | ic | | IV | + | + | 22.0 | κ |
| 6 | ic | | IV | + | − | 1.5 | — |
| 7 | ic | | IV | − | − | 1.2 | — |
| 8 | ic | κ | I | − | − | nd | — |
| 9 | ic | | II | − | − | nd | — |
| 10 | cc | λ | II | − | − | 0.61 | λ |
| 11 | cb/cc | | IV | + | + | 18.6 | κ |
| 12 | cb/cc | | IV | + | + | 8.5 | κ |
| 13 | cb/cc | | IV | + | + | 2.0 | — |
| 14 | cb/cc | | IV | + | + | 16.2 | κ |
| 15 | cb/cc | λ | IV | + | + | 0.05 | λ |
| 16 | cb/cc | | IV | + | + | 0.03 | λ |
| 17 | cb/cc | κ | IV | + | + | 5.9 | κ |
| 18 | cb/cc | | IV | − | − | 2.2 | κ |
| 19 | cb | | IV | + | + | 0.4 | λ |
| 20 | ib | | II | − | − | 1.1 | — |
| 21 | lb | | IV | − | − | 1.7 | — |
| 22 | lb | λ | II | − | − | 1.8 | — |

[a]See legend to **Table I**.

nd = Not determined; − denotes normal finding. κ:λ = ratio, κ/λ = distribution. κ or λ denotes the clonal marker in the abnormal immunological analysis.

chain analysis showed very faint but unequivocal λ labelling of all the lymphocytes. The third patient had a lymphoblastic lymphoma.

All patients with pathological κ:λ ratios also showed abnormal κ/λ profiles.

### *κ/λ distribution in primary staging (Table II)*

Five patients presented with localized disease, stage I–II. In one of these (no. 10) the κ/λ distribution in the peripheral blood was abnormal despite a normal κ:λ ratio.

Seventeen patients presented with advanced disease (stage IV). Only 4 of these were non-leukaemic according to standard methods. In one of these the κ/λ distribution analysis was abnormal, despite a normal κ:λ ratio (no. 18).

Of the patients with leukaemic lymphomas, 12 were identified in the κ/λ distribution analysis and 11 by κ:λ ratio. The patient not revealed in the immunological analyses (no. 13) has been discussed above.

### *κ/λ distribution in patients in CR (Tables III and IV)*

Thirty eight patients were in CR for a median 10 months (0–95) when the immunological analysis was performed. In 7/38 (18%) the κ/λ distribution analysis showed evidence of circulating lymphoma cells. In all these 7 patients the κ:λ ratio was normal. None of them had had lymphoma involvement of the bone marrow during their course of disease. All but one (no. 29) had low grade malignant lymphomas.

**Table III** Abnormal κ/λ distributions in relation to histopathology and tumour burden

| | Abnormal κ/λ distribution/ total number of patients: Large tumour burden (%) | Minimal disease (%) | Complete remission (%) |
|---|---|---|---|
| Low grade ml | 22/34(65) | 6/11(55) | 6/31(19) |
| High grade ml | 4/9 (44) | 1/4 (25) | 1/7 (14) |
| Total | 26/43(60) | 7/15(47) | 7/38(18) |

Low grade ml = lc, ic, cc and cb/cc.
High grade ml = lb, ib and cb.

The patients in CR were followed for a median of 17 months (1–60) after the immunological analysis

**Table IV** Twenty patients with normal routine haematology but abnormal $\kappa/\lambda$ distribution – initial staging data, data at time of sampling and follow up

| | *Initial staging data* | | | *Data at immunological analysis* | | | | |
|---|---|---|---|---|---|---|---|---|
| *Patient no.* | *Kiel class*[c] *(ml)* | *Stage* | *Marrow* | *Tumour mass* | *Therapy* | $\kappa{:}\lambda$ | $\kappa/\lambda$ | *CR, months* |
| 23 | ic | I | – | CR | – | 1.1 | $\lambda$ | 9 |
| 24 | cb/cc | II | – | CR | – | 2.2 | $\kappa$ | 10 |
| 25 | cb/cc | III | – | CR | – | 2.3 | $\kappa$ | 60 |
| 26 | cb/cc | I | – | CR | – | 2.3 | $\kappa$ | 95 |
| 27 | cb/cc | I | – | CR | – | 1.6 | $\kappa$ | 24 |
| 28 | cc | IV | – | CR | – | 1.5 | $\kappa$ | 49[a] |
| 29 | ib | III | – | CR | – | 2.7 | $\kappa$ | 7[b] |
| 30 | lc+ib | IV | + | large | – | 0.1 | $\lambda$ | |
| 31 | lc | IV | + | minimal | + | 9.0 | $\kappa$ | |
| 32 | lc | IV | + | minimal | + | 38 | $\kappa$ | |
| 33 | ic | I | – | minimal | + | 3.1 | $\kappa$ | |
| 34 | ic | IV | + | large | – | 5.1 | $\kappa$ | |
| 35 | cc | II | – | minimal | – | 0.4 | $\lambda$ | |
| 36 | cc | II | – | large | – | 0.6 | $\lambda$ | |
| 37 | cc | IV | + | minimal | + | 0.01 | $\lambda$ | |
| 38 | cb/cc | III | – | minimal | + | 4.2 | $\kappa$ | |
| 39 | cb/cc | IV | + | large | – | 0.3 | $\lambda$ | |
| 18 | cb/cc | IV | – | large | – | 2.2 | $\kappa$ | |
| 40 | cb | IV | + | large | + | 18 | $\kappa$ | |
| 41 | ib | III | – | large | + | 0.4 | $\lambda$ | |

[a]Relapse 19 and [b]3 months after sampling. [c]See legend to **Table I**. $\kappa{:}\lambda$ = ratio, $\kappa/\lambda$ = distribution, CR = complete remission.

was performed. In the group with abnormal $\kappa/\lambda$ distributions 2/7 relapsed (1 low grade ml, 1 high grade ml) compared with 7/31 in the CR group with normal $\kappa/\lambda$ distributions (5 low grade ml, 2 high grade ml).

*$\kappa/\lambda$ distribution in patients with active disease (Tables III and IV)*

This group included 22 patients analysed in primary staging and 36 patients examined at variable times after diagnosis. In 20 patients treatment with chemotherapy was ongoing.

In patients with active disease 33/58 (57%) showed abnormal $\kappa/\lambda$ patterns in the peripheral blood. Looking only at those with normal routine haematology the corresponding figures were 13/35 (37%). All but 2 were in the low grade malignant group. Eight of these 13 patients also showed abnormal $\kappa{:}\lambda$ ratios.

In patients with a large tumour burden 26/43 (60%) showed abnormal $\kappa/\lambda$ distributions. The corresponding figure in the group with minimal disease was 7/15 (47%). In patients with normal routine haematology but abnormal $\kappa/\lambda$ distribution the figures were 6/20 (30%) in the group with a large tumour burden and 7/15 (47%) in the group with minimal disease. These differences were not significant (Chi-square test, Fischer's exact test).

### Discussion

In patients with NHL of B cell type, a disturbed $\kappa{:}\lambda$ ratio, or more precisely, an abnormal $\kappa/\lambda$ distribution in the lymphocyte population in the peripheral blood, is a strong indication of circulating lymphoma cells. This is not, however, absolute proof, since light chain restriction in a population is merely evidence of monoclonal cells (Teodorescu *et al.*, 1978; Ault, 1979). Although identification of the idiotype would be the true clonal marker, this is a tedious procedure, inconvenient for routine examination.

In this study immunological classification of the lymphoma was performed in 19/96 patients. In all of these cases with a pathological $\kappa/\lambda$ distribution, the identified light chain was identical in blood and lymphoma tissue (10/19). None of the 17 patients with various other malignant or benign diseases showed abnormal $\kappa/\lambda$ patterns in their peripheral blood. Thus, even if the possibility that some other disorder might cause an immunological disturbance resulting in a shift in the κ/λ distribution cannot be ruled out, it does seem more likely that this finding, in a patient with a B cell malignancy, indicates dissemination of lymphoma cells. Another possible source of error might be the presence of monoclonal Ig in the serum of the patients. Theoretically such Ig could be passively adsorbed to the normal lymphocytes, thus simulating a monoclonal population. This does not seem to be a major drawback, when using the preincubation procedures described by Lobo *et al.* (1975). Only 8/96 patients had an M-component identified at routine screen electrophoresis, and 6 of these had quite normal κ/λ patterns in their peripheral blood.

Using the κ/λ distribution analysis, we found significantly higher proportions of patients with evidence of leukaemic spread compared to routine haematological methods – 42% *versus* 24%. These figures have to be interpreted with some caution, since routine haematological methods were not fully made use of – a blood smear was performed in one third of the patients only. Routine use of blood smears might have revealed more cases of leukaemia. To assess the magnitude of the error we have reviewed the outcome of 182 differential counts in our department in lymphoma patients with a normal wbcc. In 7% of these there was either a lymphocytosis or abnormal cells considered to be evidence of leukaemia. Even if this is taken into account, it is obvious that this immunological method to detect circulating lymphoma cells is more sensitive than routine haematology and our results are in good agreement with those of others using similar light chain distribution analyses (Ault, 1979; Ligler *et al.*, 1979). An abnormal κ/λ distribution on peripheral blood lymphocytes was a more common finding in patients with low grade malignant lymphomas. Almost two-thirds of these patients showed immunological evidence of circulating lymphoma cells. Similar results have been reported by others (Abdul-Cader *et al.*, 1983) and are consistent with the finding that these indolent lymphomas are widely disseminated (Heifetz *et al.*, 1980).

We had anticipated finding a correlation between tumour mass and presence of circulating lymphoma cells. Even if the proportion of patients with abnormal κ/λ patterns tended to be higher in the group with a large tumour burden compared to those with minimal disease, the difference was not significant. Evidently, the presence of malignant cells in the peripheral blood was related more to tumour type than tumour burden.

It is known that determination of κ:λ ratios is superior to conventional haematology for the detection of leukaemic spread in B cell lymphomas (Garrett *et al.*, 1979). We have compared the sensitivity of κ:λ ratio determinations with those of κ/λ distributions. Since κ/λ distribution analysis is in essence a light chain estimation at discrete levels of fluorescence, it would be logical to assume that the distribution analysis is more sensitive (Ligler *et al.*, 1980). When only small numbers of monoclonal cells are present, neither the total percentage nor the ratio of κ and λ positive cells will be much changed. Moreover, the normal range of the ratio is broad, which further reduces sensitivity. Our data support these ideas, since we found the κ/λ distribution analysis to be a clearly more sensitive method.

The finding of an abnormal κ/λ distribution in 18% (7/38) of the patients considered to be in CR for, in some cases, several years, is of great interest. The majority of them (6/7) had low grade malignant lymphomas. These results might be compatible with the current opinion that these lymphomas are chronic diseases with a constant relapse rate over many years (McKelvey & Moon, 1977). In 17 months (median) follow up only one of these 6 patients has relapsed. The only patient with a high grade malignant lymphoma in CR and with abnormal κ/λ distribution relapsed shortly thereafter. In the CR group without any signs of circulating lymphoma cells in the immunological analysis the relapse rate was much the same 22% (7/31). More patients and a longer period of follow up is needed before any conclusions can be drawn.

In patients with active disease and normal routine haematology the κ/λ distribution analysis yielded a diagnostic gain in 37%. We failed to identify three leukaemic samples by κ/λ distribution analysis. The first was a patient with ml cb/cc with marrow involvement but a normal blood lymphocyte count. One possible explanation might be that the cells described as abnormal in the blood smear were actually activated normal lymphocytes rather than lymphoma cells. The second patient had a CLL of B cell type, as determined by the analysis of the total amount of sIg. The amount of light chain was, however, below the detectable level, thus yielding a negative finding in the κ/λ profile (Ligler *et al.*, 1983). In the third patient, with ml ib it might be that the lymphoma was not of B cell lineage, since it is known that this group also includes T cell tumours (Habeshaw *et al.*, 1983).

The centrocytic lymphomas comprise a small group, not permitting too extensive conclusions. Nonetheless, it is our definite impression, that it is in this group that we find the most striking differences between routine haematology and κ/λ distribution analyses. This might reflect an inherent feature in the behaviour of this tumour (Swerdlow *et al.*, 1983) or might be due to methodological aspects. In our experience, centrocytes have a high

Ig density on the cell surface and are easily detected by immunological analysis, although they might be difficult to distinguish as malignant in a routine blood smear.

The conclusion to be drawn from this study is that quantitative light chain analysis is a more sensitive method of detecting circulating lymphoma cells than routine haematological methods. It is a simple and convenient test well suited for routine use in monitoring patients with NHL during treatment and in follow up. The prognostic significance of small numbers of circulating lymphoma cells in patients in CR and in stage I disease has yet to be ascertained. Further studies and long term follow up is needed.

This work was supported by grants from the John and Augusta Persson Fund for Medical Scientific Research at the University of Lund, Sweden, The Swedish Medical Research Counsil (B85-12X-05946-05A) and The Medical Faculty of the University of Lund, Sweden.

### References

ABDUL-CADER, RICHARDSON, L., WALSH, L. & 4 others. (1983). The incidence of B cell leukaemia and lymphopenia in B cell neoplasia in adults: A study using the Kiel classification of non-Hodgkin's lymphoma. *Br. J. Cancer*, **48**, 185.

AISENBERG, A.C. & LONG, J.C. (1975). Lymphocyte surface characteristics in malignant lymphoma. *Am. J. Med.*, **58**, 300.

AULT, K.A. (1979). The detection of small numbers of monoclonal B lymphocytes in the blood of patients with lymphoma. *N. Engl. J. Med.*, **300**, 1401.

GARRETT, J.V., SCARFFE, J.H. & NEWTON, R.K. (1979). Abnormal peripheral blood lymphocytes and bone marrow infiltration in non-Hodgkin's lymphoma. *Br. J. Haematol.*, **42**, 41.

GREY, H.M., RABELLINO, E. & PIROFSKY, B. (1971). Immunoglobulins on the surface of lymphocytes. IV. Distribution in hypogammaglobulinemi, cellular immunodeficiency and chronic lymphatic leukemia. *J. Clin. Invest.*, **50**, 2368

HABESHAW, J.A., BAILEY, D., STANSFELD, A.G. & GREAVES, M.F. (1983). The cellular content of non-Hodgkin lymphomas: A comprehensive analysis using monoclonal antibodies and other surface marker techniques. *Br. J. Cancer*, **47**, 327.

HEIFETZ, L.J., FULLER, L.M., RODGERS, R.W. & 5 others. (1980). Laparotomy findings in lymphangiogram-staged I and II non-Hodgkin's lymphomas. *Cancer*, **45**, 2778.

KILLANDER, D., JOHANSSON, B. & HAGLUND, S. (1977). Immunofluorometric analysis of membrane bound IgM in individual human lymphoid cells in cases of leukemia and malignant lymphoma. In *Immunological Diagnosis of Leukemia and Lymphomas*, p. 147. (Ed. Theirfeld) Springer Verlag: New York.

KUMAGAI, K., ABO, T. & SEIKIZAWA, M. (1975). Studies of surface immunoglobulins on human B lymphocytes. I. Dissociation of cell bound immunoglobulins with acid pH or at 37°C. *J. Immunol.*, **115**, 982.

LEVY, R., WARNKE, R., DORFMANN, R.F. & HAIMOVICH, J. (1977). The monoclonality of human B-cell lymphomas. *J. Exp. Med.*, **145**, 1014.

LIGLER, F.S., VITETTA, E.S., SMITH, R.G. & 4 others. (1979). An immunological approach for the detection of tumor cells in the peripheral blood of patients with malignant lymphoma; implications for the diagnosis of minimal disease. *J. Immunol.*, **123**, 1123.

LIGLER, F.S., SMITH, R.G., KETTMAN, J.R. & 5 others. (1980). Detection of tumor cells in the peripheral blood of nonleukemic patients with B-cell lymphoma: Analysis of "clonal excess". *Blood*, **55**, 792.

LIGLER, F.S., KETTMAN, J.R., SMITH, R.G. & FRENKEL, E.P. (1983). Immunoglobulin phenotype on B cells correlates with clinical stage of chronic lymphocytic leukemia. *Blood*, **62**, 256.

LOBO, P.I., WESTERVELT, F.B. & HOROWITZ, D.A. (1975). Identification of two populations of immunoglobulin-bearing lymphocytes in man. *J. Immunol.*, **114**, 116.

LUKES, R.J., TAYLOR, C.R., CHIR, B. & 5 others. (1978). A morphological and immunological surface marker study of 299 cases of non-Hodgkin lymphomas and related leukemias. *Am. J. Pathol.*, **90**, 461.

McKELVEY, E.M. & MOON, T.E. (1977). Curability of non-Hodgkin's lymphomas. *Cancer Treat. Rep.*, **61**, 1185.

PREUD'HOMME, J.L. & SELIGMANN, M. (1972). Surface bound immunoglobulins as a cell marker in human lymphoproliferative disease. *Blood*, **40**, 777.

SKLAR, J., CLEARY, M.L., THIELEMANS, K. & 3 others. (1984). Biclonal B-cell lymphomas. *N. Engl. J. Med.*, **311**, 20.

SLEASE, R.B., WISTAR, R. & SCHER, I. (1979). Surface immunoglobulin density on human peripheral blood mononuclear cells. *Blood*, **54**, 72.

SWERDLOW, S.H., HABESHAW, J.A., MURRAY, B.A. & 3 others. (1983). Centrocytic lymphomas: a distinct clinicopathologic and immunologic entity. A multiparameter study of 18 cases at diagnosis and relapse. *Am. J. Pathol.*, **113**, 181.

TEODORESCU, M. & MAYER, E.P. (1978). Surface immunoglobulin in immunoproliferative disease. *Ann. Clin. Lab. Sci.*, **8**, 353.